# First case report of *Candida guilliermondii* native left-sided valve endocarditis

**DOI:** 10.3389/fcvm.2023.1273255

**Published:** 2023-12-04

**Authors:** Zilong Zheng, Xiaokang Tu, Chuanhao Jiang, Feng Liu, Chengming Fan

**Affiliations:** ^1^ Department of Cardiovascular Surgery, The Second Xiangya Hospital, Central South University, Changsha, China; ^2^ Department of Laboratory Medicine, The Second Xiangya Hospital, Central South University, Changsha, China

**Keywords:** infective endocarditis, fungal endocarditis, cardiac surgery, mitral valve, aortic valve, *Candida guilliermondii*

## Abstract

Endocarditis, a life-threatening inflammation of the endocardium, is incited by bacteria, fungi, or other pathogenic microorganisms. Fungal endocarditis closely mirrors bacterial endocarditis in clinical signs and symptoms, leading to potential misdiagnoses. Here, we unveil the inaugural confirmed instance of native left-sided valve endocarditis attributed to *Candida guilliermondii*. Diagnosis was substantiated through valvular biopsies, blood and vegetative cultures. Treatment encompassed surgical excision of vegetations along with a six-week regimen of fluconazole administration (12 mg/kg/day), followed by 4 years of meticulous monitoring, resulting in sustained patient recovery.

## Introduction

Infective endocarditis (IE) is fraught with a 30-day morbidity and mortality rate of ∼30% (1, 2). It is noteworthy that blood culture-negative endocarditis (BCNE) may account for up to 70% of all endocarditis cases (3). The causative agents encompass bacteria, fungi, and other pathogenic microorganisms that invade the circulatory system, leading to endocardial and cardiac valve aggregation, often resulting in vegetative formations. Although common causative factors include pathogens such as coagulase-negative Staphylococci, Staphylococcus aureus, and Enterococcus spp. (4), fungal endocarditis remains infrequent, constituting only 2%–4% of all IE cases (5). Nevertheless, it is one of the most severe forms of infective endocarditis (6), with a documented in-hospital mortality rate of 16.2% (7). Fungal endocarditis can present as native valve endocarditis, prosthetic valve endocarditis, inflammation of the endocardial surface, or cardiac device-related infective endocarditis (8). The frequency of fungal native-valve endocarditis is largely unknown, however this disease affects nearly 0.1% of all prosthetic cardiac valves (9). Despite its rarity, Fungal endocarditis can present in any isolated heart valve or the combination of them (10–13). Rising fungal endocarditis cases are attributed to improved diagnostic techniques, usage of immunosuppressants, invasive procedures, and the prevalent use of central venous catheters, which escalate the risk of medical-related IE. The efficacy of surgical and antifungal interventions plays a pivotal role in managing complicated IE (7). Notably, Candida species predominate fungal endocarditis constituting 46% of cases, followed by Aspergillus and Histoplasma spp. (14, 15). At least 30 Candida species have been recognized as causes of human infection, and the list continues to expand. Advances in identification and taxonomy of yeast have led to the recognition of many novel cryptic species (16). However, *Candida guilliermondii* infection affecting native left-side cardiac valves remains unreported. We present an unprecedented instance of severe native mitral and aortic valve endocarditis due to *Candida guilliermondii*, emphasizing the successful interplay of antifungal therapy and surgical intervention.

## Case presentation

A 53-year-old female patient presented to our cardiac center with a history of prolonged fever and persistent chest distress spanning the past 7 months. Notably, she denied any pertinent family history of cardiovascular ailments and exhibited no records of trauma, smoking, or drug abuse. Furthermore, there was no history of rheumatic fever. The patient denied the risk factors associated with Candida spp. infection including immunocompromised states. She had a medical background characterized by hypertension, ovarian cyst, and an isolated right temporal lobe stroke. Before admission, and in the local hospital, this patient received empiric antibiotics (ceftriaxone, 2 g ivgtt qd) for 7 days. Upon admission, her body temperature was recorded at 36.2°C, accompanied by a blood pressure of 162/52 mmHg, a heart rate of 94 bpm, and a radial pulse rate.

**Figure 1:**
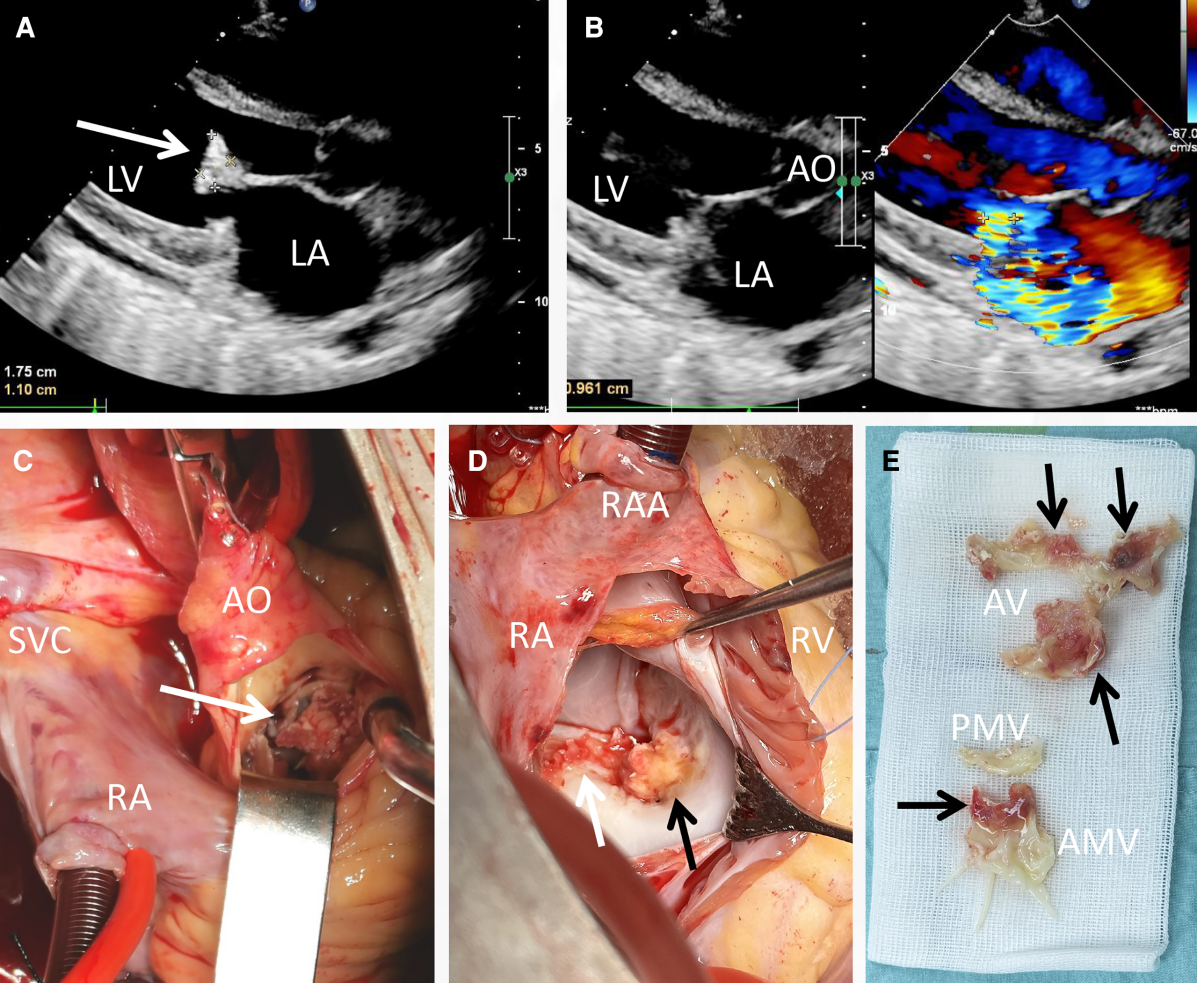
Transthoracic echocardiogram and intraoperative view of the vegetations. A vegetation sizing 1.75 cm × 1.10 cm (**A**, arrow), as well as severe mitral regurgitation (**B**) were detected pre-operatively; the yellowish vegetation (arrow) was observed intra-operatively with the infiltration of aortic valve (**C**), anterior mitral cusp (**D**) and mitral annulus (**E**).

Laboratory investigations, including routine blood tests, unveiled a leukocyte count of 1.91 × 10^9^/L, with an absolute neutrophil count of 1.08 × 10^9^/L. Hemoglobin levels were measured at 88 g/L, NT-proBNP at 2,697 pg/ml, C reactive protein at 27 mg/L and the erythrocyte sedimentation rate at 53 mm/h. The (1,3)-β-d-glucan (BDG) and galactomannan (GM) tests were both detected positive. During physical examination, a pronounced precordial murmur was discernible during both systole and diastole. Notably, the chest radiograph did not exhibit significant signs of abnormality, except for a mild enlargement of the cardiac silhouette. Transthoracic echocardiogram (TTE) assessment (on the day of admission and 1 day before the operation) unveiled numerous echogenic vegetations affixed to the anterior mitral valve, including one particularly prominent vegetation measuring 17 × 11 mm (Figure 1A, arrow). This echocardiogram also indicated severe mitral insufficiency (Figure 1B). No embolic lesions were detected in both the cranial CT scanning and abdominal echography.

Based on the findings from echocardiography, the BDG test and GM test results, the diagnosis of fungal endocarditis was strongly considered according to the DUKE criteria. A further detection though transesophageal echography was refused by the patient. Consequently, a valve replacement procedure was undertaken (1 day after the admission). Direct intracardiac surgery was conducted through a median sternotomy, with extracorporeal circulation routinely established through aortic, superior vena cava, and inferior vena cava cannulation at a mild hypothermia of 30°C. During the operation, additional small vegetations were detected on the aortic valve (2–4 mm in diameter), along with perforation (3 mm in diameter) of the valve cusp (Figure 1C, arrow). The infective valvular vegetation, displaying a yellowish hue, was approached via the ascending aorta (Figure 1C) and interatrial septum (Figure 1D). The vegetation was successfully excised (Figure 1E), and subsequent replacement with two mechanical valves (Sorin, sizing 25 mm and 27 mm for aortic and mitral valves, respectively) was carried out. The operation and postoperative recovery proceeded without complications. The post-operative data including blood routine and myocardial zymology were shown in supplement Tables 1, 2. The symptoms including the fever and persistent chest distress were not found post-operatively.

**Table 1:** Change of blood routine post-operatively.

	PO-day 11	PO-day 9	PO-day 7	PO-day 5	PO-day 3	PO-day 1
Hemoglobin (g/L)	100	91	115	105	110	99
PLT (no.)	250	225	260	219	214	208
RBC (no.)	3.51	3.07	3.92	3.50	3.80	3.50
WBC (no.)	9.36	11.29	12.63	16.09	13.65	11.65
HCT (%)	32.0	28.5	36.9	32.1	35.1	30.9
Neutrophil (no.)	7.29	9.70	10.96	15.48	12.98	10.29
Neutrophil ratio (%)	77.9	86.0	86.7	96.1	95.1	88.3

**Table 2:** Change of myocardial zymology post-operatively.

	PO-day 11	PO-day 9	PO-day 6	PO-day 3
cTnI (μg/L)	0.64	1.09	2.92	2.37
D-dimer (mg/L)	2.11	1.16	1.89	4.02
Myoglobin (ng/ml)	8.1	22.5	87.6	>500
CK-MB (U/L)	<2.00	15.2	59.3	>100
NT-proBNP (pg/ml)	426	3,521	4,856	8,625

Pre-operative blood cultures and biopsies (valvular tissue, intra-operatively) were instrumental in identifying *Candida guilliermondii*, affirming the previous diagnosis and indicating sensitivity to the prescribed antifungal regimen, including fluconazole. Thus, antifungal treatment with fluconazole (12 mg/kg/day) was given post-operatively. Subsequently, the patient was transferred from the cardiac intensive care unit and discharged from the hospital on the 11th postoperative day. Notably, three blood cultures performed prior to discharge yielded negative results. The patient was then referred to the local Department of Cardiology for a continued six-week antifungal treatment (fluconazole, 12 mg/kg/day, po). Over the ensuing 4-year follow-up period, the patient exhibited a complete recovery, remaining devoid of any symptomatology.

## Discussion and conclusion

Candidemia stands as a potentially life-threatening fungal infection primarily afflicting patients with prolonged intravenous catheter use, hemodialysis, hematopoietic stem cell transplantation, and profound immune deficiency (17). *Candida guilliermondii*, a constituent of the human microbiota, seldom emerges as a pathogenic agent, underscoring its infrequent identification in patient infections. Throughout the years, this fungus has been mostly collected within cancer and haematology wards, especially in the patients with eating disorders (17, 18). Another relatively common group is from dermatology services and the organ transplant service (19, 20). Owing to its low prevalence, the exploration of *Candida guilliermondii* infections lags behind that of more prominent candida species (21). Theoretically, invasion of circulation by *Candida guilliermondii,* leading to endocardial erosion or cluster formation on cardiac valves, can incite IE. Nevertheless, left-side valve IE prompted by *Candida guilliermondii* remains unprecedented. Given the heightened risk of embolism and hemorrhagic complications within fungal endocarditis, Ellis and colleagues reported an incidence rate of 45%, with cerebral thromboembolism being the most frequent complication (7). Consequently, the imperative for timely diagnosis becomes evident.

In instances of suspected valvular endocarditis, echocardiography emerges as the preferred diagnostic modality, enabling the noninvasive assessment of vegetation morphology, size, mobility, impact on cardiac function, interaction with neighboring tissues, and valve severity (22). For patients with positive Candida blood cultures, echocardiography is particularly recommended. While the diagnosis and causative microorganism of IE are confirmed, the Infectious Diseases Society of America's 2016 candidiasis guidelines advocate a dual approach encompassing valve replacement and prolonged antifungal therapy for the management of Candida endocarditis (7). Nonetheless, addressing active complex IE through surgical intervention remains a challenge, associated with elevated rates of operative morbidity and mortality (23). The mounting resistance to antifungal agents due to unregulated and improper antimicrobial usage further compounds this challenge (21). The combination of fluconazole with one or more other antifungal treatments has demonstrated efficacy in certain instances of Candida endocarditis. Nonetheless, employing fluconazole as a standalone initial therapy for Candida endocarditis has been linked to unfavorable outcomes (24). For the patients managed with fluconazole-containing antifungal therapy plus valvular surgery, survival was 91% (24). Hence, the spectrum of IE treatment spans from antimicrobial therapy and valvular repairs/replacement to potential heart transplantation (23, 25).

In this context, the strength of the case presented lies in being the inaugural documentation of severe fungal infective endocarditis involving native left-sided valves, attributed to *Candida guilliermondii*. Significantly, the patient exhibited a favorable outcome following timely valvular replacement and targeted antifungal therapy. The case underscores the importance of considering fungal endocarditis when empirical antibiotic therapy fails to yield improvement, especially when the BDG test and GM test were positive. Nevertheless, as a case study, it should be regarded as preliminary evidence and not the sole basis for clinical practice or policy decisions. Researchers and readers should approach case reports with caution and within the broader context of medical evidence. In conclusion, the case emphasizes the efficacy of a comprehensive and multidisciplinary approach involving prompt diagnosis, adaptive antimicrobial strategies, and precise surgical intervention. This integrated strategy proves invaluable in managing this challenging clinical entity by minimizing mortality rates and enhancing patient prognoses.

## Data Availability

The raw data supporting the conclusions of this article will be made available by the authors, without undue reservation.
